# Strength, Durability, and Microscopic Analysis of Silt Solidified with Two-Phase Phosphogypsum and Cement Fiber

**DOI:** 10.3390/ma18091960

**Published:** 2025-04-25

**Authors:** Xiaoya Bian, Junjian Xia, Hui Liu, Tianyu Xiao

**Affiliations:** School of Civil Engineering and Architecture, Wuhan Institute of Technology, Wuhan 430074, China

**Keywords:** phosphogypsum, two-phase, cement, silt, fiber, synergistic solidified

## Abstract

The accumulation of silty soils and industrial solid waste not only results in a significant waste of land resources but also causes environmental pollution. Phosphogypsum and cement are commonly utilized as binding agents for the solidification of silt in engineering applications. However, the use of PG and cement alone may lead to issues such as insufficient strength, crack formation, and poor durability. Therefore, this research considered and employed a two-phase stabilization method using phosphogypsum and cement to solidify silt. Additionally, to further enhance the durability of the stabilized silt, polypropylene fiber (PP) and sodium sulfate (Na_2_SO_4_, NS) were incorporated. The effects of two-phase phosphogypsum and the proportion of hemihydrate phosphogypsum (BHPG) in the two-phase phosphogypsum on the strength characteristics of the stabilized silt were investigated through unconfined compressive strength tests and durability tests. The results show that when the content of two-phase phosphogypsum is 5%, and the proportion of BHPG in the two-phase phosphogypsum is 20%, the 28-day unconfined compressive strength of the stabilized silt reaches 1.42 MPa, and the deformation modulus is 95.5 MPa. After incorporating sodium sulfate (NS), the water and frost resistance of the stabilized silt significantly improved. The microstructural analysis shows that NS promotes the formation of ettringite. Furthermore, an excessively high proportion of hemihydrate phosphogypsum (BHPG) in the two-phase phosphogypsum content can lead to dihydrate phosphogypsum (2HPG) not being encapsulated by hydration products, which results in a less dense structure of the solidified silt and a decline in performance.

## 1. Introduction

Large-scale dredging projects generate substantial amounts of dredged silt, which often poses significant environmental and disposal challenges [1,2,3]. These dredged silts have high water content and low strength, which are difficult to process directly [4,5,6]. As a result, these materials occupy and waste land resources. Similarly, engineering activities such as municipal infrastructure and road construction commonly generate large amounts of excavated soil in regions with widespread soft soil distribution. These soils often contain undisturbed engineering mud-like soils, which share characteristics similar to those of dredged silt. Typically, they exhibit issues such as low strength, high compressibility, and poor permeability. The dual challenges of soil waste management and industrial byproduct utilization necessitate the development of integrated solutions. As a byproduct of wet-process phosphoric acid production, phosphogypsum generates 4–6 tons per ton of phosphoric acid [7,8]. Global stockpiles have reached 6 billion tons, with annual increments of 10–28 million tons [9,10]. This significant accumulation of phosphogypsum presents serious challenges in terms of storage, environmental impact, and sustainable disposal. At present, most phosphogypsum is treated using storage methods due to its low comprehensive utilization rate, which presents a critical threat to the surrounding environment [11].

The stabilization of large volumes of silty soil into usable engineering materials has gained widespread application due to its advantages in energy conservation, environmental protection, and high construction efficiency. At present, physical and chemical stabilization methods are the primary techniques for treating silty soil in engineering practice. Among them, the vacuum preloading method is widely used within the realm of physical treatment techniques [12,13,14]. This method effectively reduces the water content of silty soil, promotes its solidification, and improves its properties with low costs. However, its efficacy is limited for deeper layers of silty soil, which imposes certain restrictions on its application in some engineering projects. On the other hand, chemical stabilization methods predominantly use cement as the solidifying agent for silty soil [15,16]. Though cement can significantly improve the properties of silty soil and has a wide range of application scenarios, it is associated with high costs, substantial energy consumption, and environmental pollution, which evidently contradict the principles of sustainable and green development. Therefore, researchers have begun exploring the incorporation of other industrial waste-based solidifying agents into cement-stabilized soil to reduce cement usage, which has achieved certain success in the stabilization of silty soil. Liu et al. [17] added cement, fly ash, and slag as stabilizing agents in silty soil and found that the addition of fly ash and slag improved the strength of the stabilized soil.

The primary component of phosphogypsum is calcium sulfate dihydrate (CaSO_4_·2H_2_O), along with minor quantities of phosphoric acid, phosphate minerals, fluorides, and organic materials. Phosphogypsum has potential utilization value. It was found that phosphogypsum can significantly enhance the load-bearing capacity of roads [18]. Furthermore, the combined stabilization of silty soil using phosphogypsum and cement effectively improves the mechanical properties of the soil [19]. Zeng et al. [20] incorporated phosphogypsum into cement-stabilized silty soil to investigate its effects on the physicochemical properties and mechanical performance of the soil. It was found that with the increasing content of phosphogypsum, the water content and pH value decreased with the density. An appropriate amount of phosphogypsum enhanced structural densification. However, excess phosphogypsum could lead to an increase in volume and structural damage, ultimately resulting in a reduction in strength. Ou et al. [21] utilized phosphogypsum as the primary raw material in combination with a cement stabilizer for the solidification of silty soil. They discovered a method to prepare a pavement base material with excellent physical and mechanical properties, as well as durability, which can significantly enhance the load-bearing capacity of roads. Bian et al. [22] studied the engineering properties of cement–phosphogypsum solidified dredged silt using unconfined compressive strength tests. However, most of the aforementioned studies have primarily concentrated on single-phase systems (such as the independent application of dihydrous phosphogypsum or hemihydrate phosphogypsum), while the synergistic effects within two-phase composite systems remain to be systematically explored. This study transcends the conventional limitation of phase singularity in phosphogypsum research and innovatively establishes a theoretical framework for the synergistic phase interaction of dihydrous- hemihydrate complexes. It is noteworthy that industrially produced phosphogypsum typically exists in two forms: the two-aqueous phase (2HPG) and the semi-aqueous phase (BHPG) [23]. BHPG is used in the preparation of building gypsum powder. However, it readily absorbs moisture in the air and converts into 2HPG. This conversion leads to a decrease in performance. Ultimately, it becomes a mixture of dihydrate and hemihydrate gypsum [24]. Furthermore, the stabilized silty soil encounters problems such as brittle failure and inadequate water stability, which complicate its ability to meet long-term application demands in complex environments [25]. To address these issues, previous studies have shown that adding chemical admixtures and reinforced fibers can effectively improve both the curing performance and long-term durability of the stabilized silty soil [26,27,28].

To address the aforementioned issues, this study proposes the use of two-phase phosphogypsum in conjunction with cement to stabilize silty soil. Additionally, the performance is further optimized by incorporating chemical additives (Na_2_SO_4_) and polypropylene fibers. The effects of varying contents and component ratios of two-phase phosphogypsum on the strength of stabilized silt through unconfined compressive strength tests are investigated, and water resistance tests and freeze–thaw cycle tests are conducted to evaluate the enhancing effect of sodium sulfate on the durability (water resistance and frost resistance) of stabilized silt. Meanwhile, the stabilization mechanism of the two-phase phosphogypsum in conjunction with cement is discussed using microstructural analysis.

## 2. Research Program

**Figure 1:**
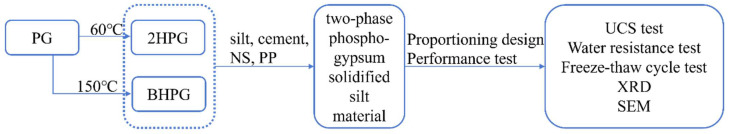
The research workflow of this study.

The two-phase phosphogypsum solidified soil material is composed of cement, polypropylene fibers, NS, and phosphogypsum silt soil. In this study, the properties of the two-phase phosphogypsum solidified material were systematically investigated through proportioning design, unconfined compressive strength testing, water resistance evaluation, and freeze–thaw cycle analysis. Figure 1 illustrates the research workflow of this study.

## 3. Materials and Methods

### 3.1. Materials

**Figure 2:**
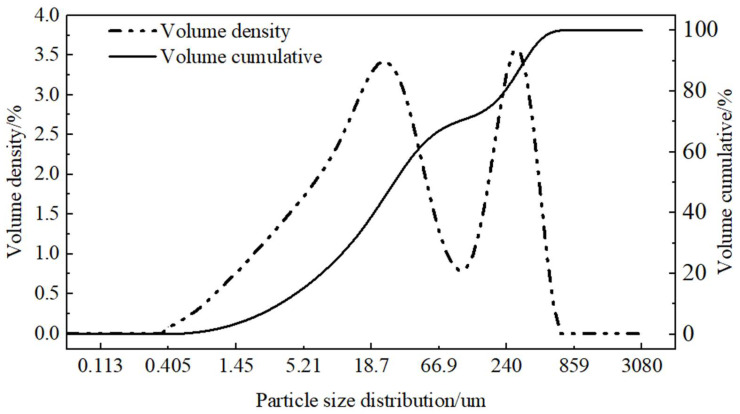
Particle size distribution of test soil.

This experiment utilized soft soil collected from a construction site in Wuhan to prepare the silty soil samples. The soft soil exhibited a reddish-brown coloration. The basic physical properties of the soil are presented in Table 1, and the particle size distribution is illustrated in Figure 2. The liquid and plastic limits were determined using the conical liquid–plastic limit combined tester method according to the Highway Geotechnical Test Procedures (JTG 3430-2020-T0018-2007). The results classified the experimental soil as low-liquid–plastic-limit red clay based on the liquid–plastic limit.

**Figure 3:**
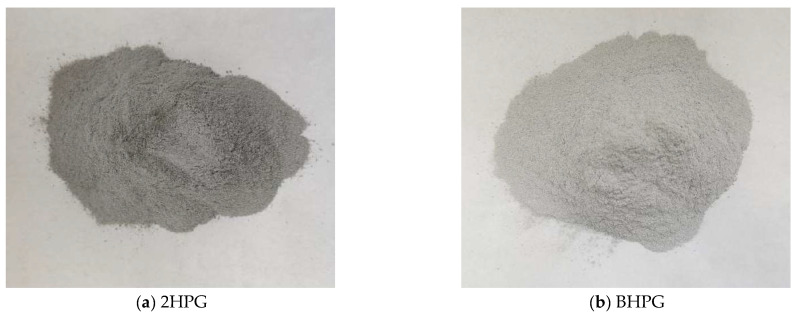
Phosphogypsum.

**Figure 4:**
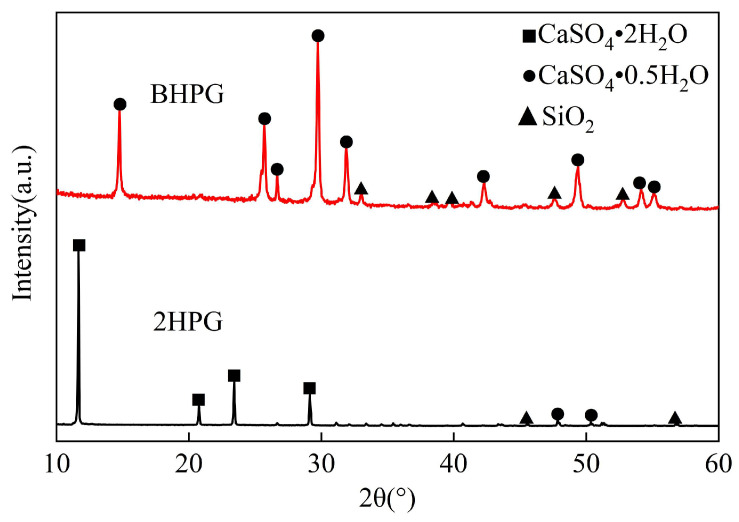
XRD pattern of 2HPG and BHPG.

The phosphogypsum used in this study was sourced from a chemical plant in Yichang, Hubei Province. It is a gray-black powder with crystalline particles and exhibits weak acidity. The natural moisture content of the phosphogypsum is 23.6%. Table 2 presents the chemical compositions of the phosphogypsum. Phosphogypsum subjected to low-temperature treatment (60 °C) can be converted into dihydrate phosphogypsum (2HPG, CaSO_4_·2H_2_O), which appears gray-black (Figure 3a). When phosphogypsum undergoes high-temperature treatment (150 °C), it achieves an 85% conversion rate to hemihydrate phosphogypsum (BHPG, CaSO_4_·0.5H_2_O), which appears gray-white (Figure 3b). The two-phase phosphogypsum described in this study comprises a specific mixture of dihydrate phosphogypsum (2HPG) and hemihydrate phosphogypsum (BHPG) in a predetermined proportion. The mineral composition of phosphogypsum is analyzed through X-ray diffraction (XRD), as illustrated in Figure 4.

This study used P.O. 42.5 ordinary Portland cement provided by Huaxin Cement; it primarily consists of CaO and SiO_2_. The chemical composition is detailed in Table 3.

In order to obtain engineering materials with superior comprehensive properties, this study incorporates chemical additives and fibers to further enhance the characteristics of solidified silt. The study used polypropylene fiber (PP) produced by a fiber supply factory in Hubei. The fibers have a circular cross-section, a smooth surface, and a length of 9 mm. Their physical and mechanical properties are presented in Table 4. The chemical additive used in this study is analytical-grade anhydrous sodium sulfate (Na_2_SO_4_, NS), which appears as white crystalline solids and is readily soluble in water.

### 3.2. Mixture Proportion

Five experimental conditions were set up to investigate the effect of two-phase phosphogypsum and the proportion of hemihydrate phosphogypsum (BHPG) on the strength of cured silt. In the experiments, the cement content (the mass ratio of two-phase phosphogypsum and soil) was 15%, the polypropylene fiber (PP) content (the mass ratio of two-phase phosphogypsum and soil) was 0.5%, and the NS content was set at 7% (the mass ratio of cement) by mass of cement. The combined mass of two-phase phosphogypsum and soil is equal to 100%. These proportions were established based on preliminary experiments and relevant research findings [27,28]. The experiment was designed with three curing ages: 7 days, 14 days, and 28 days. The specific mix design is detailed in Table 5.

### 3.3. Sample Preparation

The sample preparation processes for this study are as follows:

(1) Preparation of silty soil: The test soil is soaked in water until saturated, and the silty soil is prepared with a moisture content of 75%.
(2) Material mixing: The PP is pre-crushed, and the cement is mixed with NS to form a slurry at a water-to-cement proportion of 1:0.5, following the Design Code for Cement–Soil Mixture Proportions (JGJT 233-2011-5.0.3). Finally, the slurry is mixed with PP and silty soil according to the specified proportions using a mini electric mixer produced by Guangdong CHIGO Instrument Co., Ltd. for 10 min.
(3) Molding: The inner walls of the mold are evenly coated with petroleum jelly to facilitate demolding. The mold is filled in three layers, thoroughly compacting each layer to expel air bubbles and ensure the sample is dense.
(4) Demolding and curing: The sample is allowed to cure for approximately 12 h and then demolded. The demolded sample is wrapped in preservative film and placed in a sealed bag. Then, it is stored in a curing chamber at a temperature of (25 ± 2) °C and humidity greater than 90% until the specified curing age is reached.

### 3.4. Test Methods

#### 3.4.1. Unconfined Compressive Strength Tests

In practical engineering, unconfined compressive strength serves as a crucial indicator for evaluating solidified soil properties. This property is particularly valuable because it can directly and intuitively reflect the structural characteristics of the material. According to the Standard for Highway Geotechnical Testing Methods (JTG 3430-2020-T0048-1993), the effectiveness of silt solidification is evaluated through unconfined compressive strength tests.

The study introduces the deformation modulus as an analytical parameter to analyze the deformation characteristics of solidified silt. The deformation modulus *E*_50_ is the secant modulus corresponding to 50% of the peak stress. It reflects the ability of material to resist elastic-plastic deformation. The deformation modulus can be calculated using the following formula:

(1) $$E_{50}=\frac{2\sigma_{1/2}}{\varepsilon_{f}}$$

where *σ*_1/2_ represents the axial stress when the failure strain reaches 1/2; *ε_f_* represents the value of strain when the sample fails.

The experiment utilized the TSZ-1A fully automatic triaxial apparatus produced by Nanjing Ningxi Soil Instrument Co., Ltd. to test the unconfined compressive strength, and the compression rate was set to 1 mm/min. The sample mold specifications had an inner diameter of 39.1 mm and a height of 80 mm. Three parallel specimens were prepared for each group of test blocks. After the experiment, outliers were removed, and the average value of the remaining data was taken as the final result.

#### 3.4.2. Water Resistance Tests

The water resistance test is a common method for evaluating the water resistance performance of solidified silt. It is primarily assessed using two indicators: the water absorption rate and the softening coefficient [29].

(1) Water absorption rate

A solidified silt sample that had been cured for 28 days was placed in a 60 °C oven for drying for 24 h until a constant weight was achieved, with the dry soil mass recorded as *M*_1_. The sample was then immersed in water for 24 h until it reached a constant weight, with the saturated soil mass recorded as *M*_2_. The water absorption rate (*W*_a_) can be calculated using the following formula:

(2) $$W_{a}=\frac{M_{2}-M_{1}}{M_{1}}$$

(2) Softening coefficient

To determine the softening coefficient of cured silt, a sample of cured silt that had been cured for 28 days was placed in a drying oven at 60 °C for 24 h until it reached a constant weight. Its unconfined compressive strength was then measured and recorded as *σ*_1_. The dried sample was subsequently immersed in water for 24 h until it reached a constant weight again, after which its unconfined compressive strength was measured once more; it was recorded as *σ*_2_. The softening coefficient was defined as the proportion of the strength after immersion to the strength after drying; it was expressed as *S*_c_ = *σ*_2_*/σ*_1_.

#### 3.4.3. Freeze–Thaw Cycle Tests

According to the Testing Specification for Inorganic Binder-Stabilized Materials in Highway Engineering (JTG E51-2009-T058), samples that have been cured for 28 days undergo water-saturated curing on the last day of the curing period. After water saturation, the samples are removed and placed in a low-temperature testing chamber at −18 °C to freeze for 16 h. Following this, the samples are thawed in a water tank at 25 °C for 8 h. This step finalizes a single freeze–thaw cycle. This process is repeated for 15 freeze–thaw cycles, with the unconfined compressive strength measured after each cycle.

The freeze–thaw resistance of the stabilized silt is evaluated using the freeze–thaw index (*BDR*), calculated using the following specific formula:

(3) $$BDR=\frac{R_{n}}{R_{c}}\times 100$$

where *BDR* represents the compressive strength loss (%) of the specimen after *n* cycles of freeze–thaw; *R_n_* is the compressive strength of the specimen after *n* cycles of freeze–thaw; *R*_c_ is the compressive strength of the control specimen.

#### 3.4.4. X-Ray Diffraction (XRD) Analysis

The experimental raw materials were dried in an oven at 45 °C for 48 h. Subsequently, the sediment samples were cured over a 28-day period. Following this, anhydrous ethanol immersion (24 h) was applied to the cured specimens to terminate residual chemical activity. The treated samples were then ground to a powder that could pass through a 0.075 mm sieve, which was used for XRD testing.

The experiment used a D8 ADVANCE X-ray diffractometer produced by German Bruker Instruments Company with a scanning angle range of 2θ from 10° to 60°. The sample scanning time was set to 30 min to investigate the mineral composition of the raw materials and the mineral formation in the cured sediment.

#### 3.4.5. Scanning Electron Microscope (SEM) Observation

The cured silt samples that had been subjected to a 28-day maintenance period were selected for SEM testing. The samples were initially immersed in anhydrous ethanol for 24 h to prevent further reactions. The flat fracture surfaces of the samples (with dimensions smaller than 5 mm^3^) were then subjected to vacuum drying at 45 °C for 48 h, followed by a gold-sputtering treatment on the dried surfaces prior to testing.

The experiment utilizes a GeminiSEM 300 model scanning electron microscope produced by German Carl Zeiss Company, with a controlled scanning speed of 2°/min, a scanning range of 5° to 80°, and a magnification of 5000 times, which is employed to observe the microstructure of the cured silt.

## 4. Result and Discussion

### 4.1. Unconfined Compressive Strength

**Figure 5:**
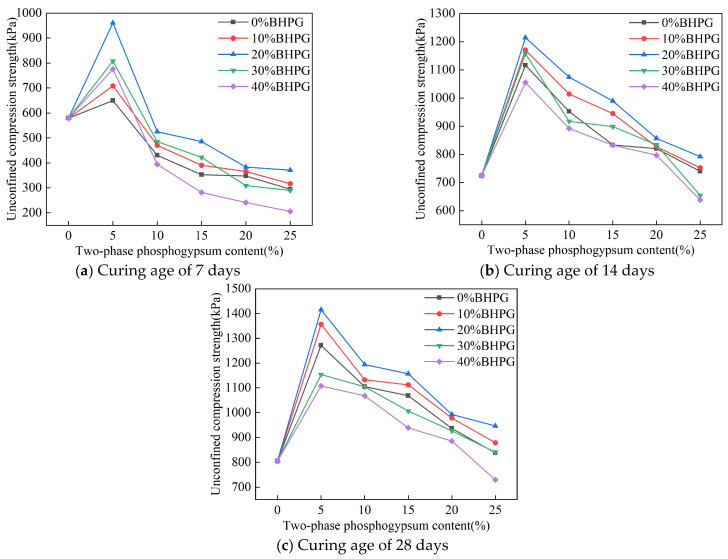
The effect of different two-phase phosphogypsum contents on the strength of solidified silt.

Figure 5 illustrates the effect of varying proportions of two-phase phosphogypsum on the unconfined compressive strength of the solidified silt. As shown in the figure, the strength of the solidified silt first increases and then decreases with the increase in two-phase phosphogypsum content and reaches a peak at a proportion of 5%. The peak strengths at curing ages of 7, 14, and 28 days are 0.96 MPa, 1.21 MPa, and 1.42 MPa, respectively. By comparing the existing literature with previous studies by Zeng [20], andBian et al. [22], it can be seen that the strength of the single-phase phosphogypsum solidified silt in this study is weaker than that of the two-phase phosphogypsum solidified silt. This can be attributed to the synergistic effect between the two phases of phosphogypsum. When the proportion is below 5%, the strength increases as the proportion rises. However, once the proportion exceeds 5%, further increases in the two-phase phosphogypsum ratio lead to a decline in strength. At a proportion of 25%, the strength is lower than that of samples without phosphogypsum. Additionally, Figure 5 indicates that the strength of solidified silt exhibits an initial increase followed by a subsequent decrease with varying BHPG content. The peak strength occurs at 20% BHPG content, and the lowest strength is observed at 40% BHPG content.

These results indicate that there is a threshold for the proportion of two-phase phosphogypsum. When the proportion is too high, the excessive formation of ettringite can cause sample expansion and structural damage. At the same time, the inert and acidic properties of phosphogypsum, when present in excess, lower the pH of the system. This inhibits the hydration reactions and consequently reduces the strength [30].

After a suitable quantity of BHPG has been converted to 2HPG, it is subsequently coated with stable hydration products, including calcium sulfoaluminate and ettringite, which enhance the structural compactness and improve the strength. However, when the BHPG content exceeds 20%, excess 2HPG remains unreacted. This limitation restricts its contribution to the structural enhancement, which ultimately leads to a decrease in strength.

### 4.2. Analysis of Failure Forms

**Figure 6:**
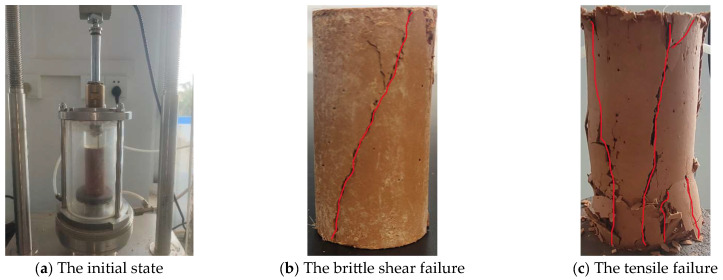
The pressurization process of solidified silt.

Figure 6 illustrates the pressurization process of solidified silt. Figure 6a illustrates the initial state of solidified silt. When the strength of solidified silt is low, the failure form of the sample, as shown in Figure 6b, is a diagonal crack running through the sample in the diagonal direction; the main crack is at a certain angle to the central axis of the sample, and the shear failure surface is clearly visible, it is brittle shear failure. When the strength of the sample is relatively high, the failure mode of the sample, as shown in Figure 6c, shows that one or more cracks along the central axis of the sample run through the entire sample, and the sides of both ends of the sample are accompanied by laminar debris shedding and small cracks of different degrees. It is a tensile failure.

### 4.3. Stress–Strain Curve

**Figure 7:**
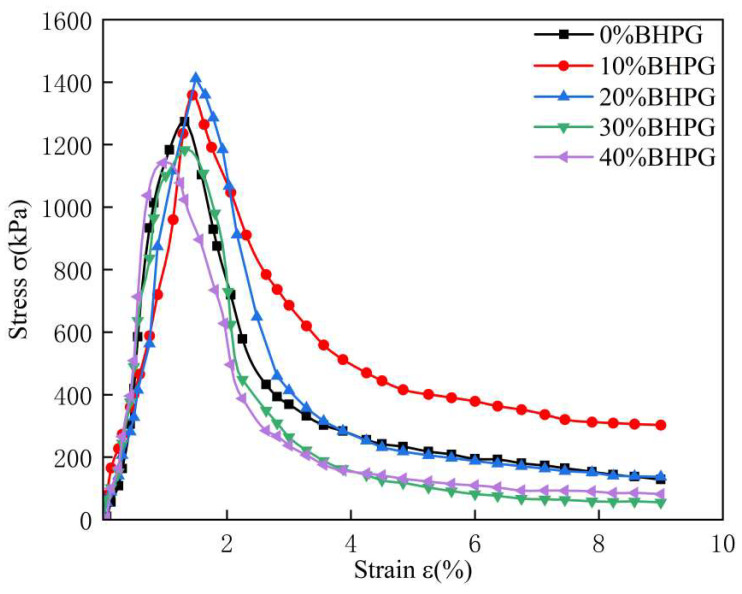
The effect of different BHPG contents on the stress–strain curve of solidified silt.

Figure 7 illustrates the impact of different BHPG contents on the stress–strain characteristics of cured sediments after a curing period of 28 days. As shown in the figure, with the BHPG content increasing, both the peak strength and failure strain of the cured sediments exhibit a trend of initially increasing and then decreasing. The stress–strain curves demonstrate strain-softening characteristics and brittle failure, with peak strength occurring around axial strains of 1% to 2%. After reaching the peak, the axial stress rapidly decreases and gradually stabilizes.

The results indicate that a BHPG content of 20% yields maximum values for both strain and strength, with the most pronounced degree of brittle failure in the cured sediments. This occurs because at BHPG contents below 20%, the increase promotes the formation of cementitious phases, which enhances the bonding forces between particles. This improved bonding leads to a more stable sample structure, thereby increasing its strength. However, when the BHPG content exceeds 20%, an excessive amount of unreacted 2HPG remains in the system. These unreacted particles offer minimal structural enhancement, which results in both a decrease in strength and an increased tendency towards brittle failure.

### 4.4. Modulus of Deformation

**Figure 8:**
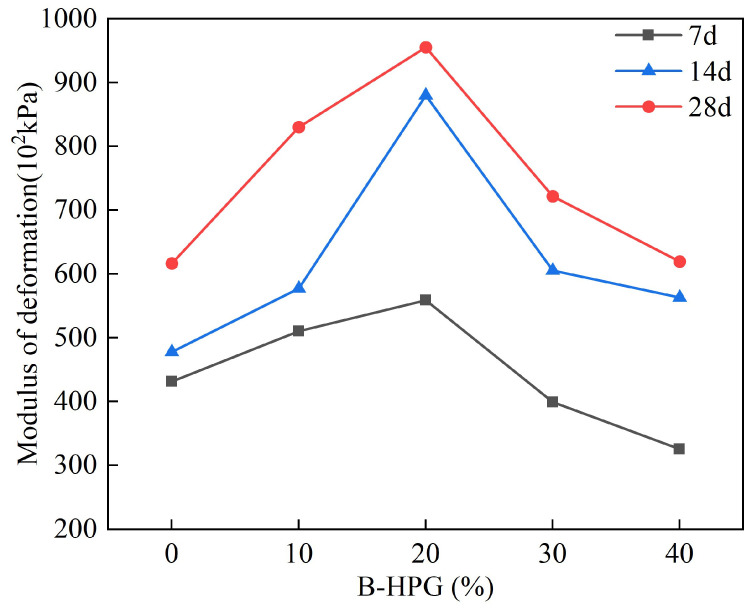
The influence of different BHPG contents on the deformation modulus of solidified silt.

Figure 8 illustrates the impact of different BHPG contents on the deformation modulus of solidified sediments. As shown, the deformation modulus initially increases and then decreases with rising BHPG content, and it reaches a peak at 20% BHPG with a modulus of 95.5 MPa after 28 days of curing. When the BHPG content exceeds 20%, the deformation modulus gradually declines. Furthermore, the deformation modulus tends to increase overall with more extended curing periods.

The results indicate that a BHPG content of 20% achieves the optimal integrity and structural stability of the solidified sediment. This achieves the best resistance to deformation. This is attributed to a strong linear relationship between the deformation modulus and the unconfined compressive strength, both reaching maximum values at 20% BHPG content.

### 4.5. Water Resistance

**Figure 9:**
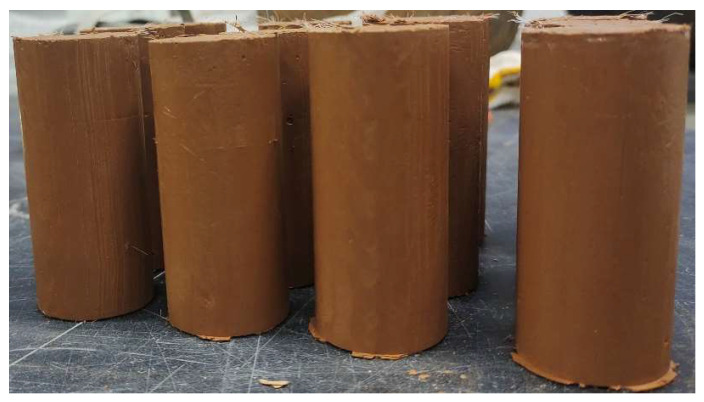
The specimen characterization diagram after curing for 28 days.

**Figure 10:**
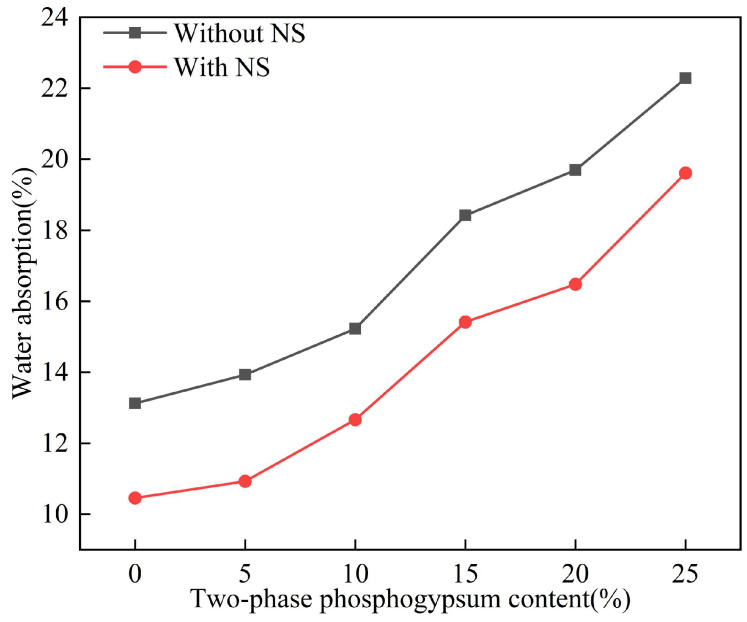
The effect of two-phase phosphogypsum content on the water absorption of solidified silt.

Figure 9 illustrates the specimen characterization diagram after curing for 28d, and Figure 10 illustrates the effect of two-phase phosphogypsum content on the water absorption rate of solidified silt. As the phosphogypsum content increases, the water absorption rate of the solidified silt gradually rises. When the phosphogypsum content increases from 0% to 25%, the water absorption rates without NS and with NS increased by 69.7% and 87.58%, respectively. The increase in water absorption is relatively slow when the content rises from 0% to 5%, but it sharply escalates with further additions.

After the addition of the additive NS, the water absorption rate of the solidified silt exhibited a significant decrease, with the reduction ranging from 13.6% to 27.4%. The highest water absorption rate was reduced to 19.61%, and the optimal NS content was determined to be 5%, at which the water absorption rate was 10.45%.

**Figure 11:**
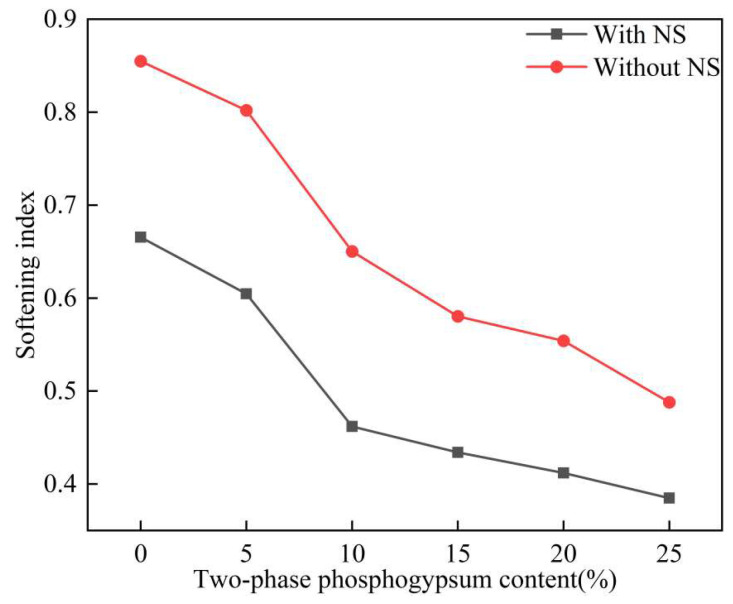
The effect of two-phase phosphogypsum content on the softening coefficient of solidified silt.

Figure 11 illustrates the impact of the two-phase phosphogypsum content on the softening coefficient of solidified silt. As the two-phase phosphogypsum content increases, the softening coefficient of the solidified silt gradually decreases. This indicates that a reduction in the water resistance of the material occurred. When the content rises from 0% to 25%, the softening coefficients without NS and with NS decline by 72.9% and 76.9%, respectively.

After the addition of the additive NS, the softening coefficient of the solidified silt significantly increases. The improvement ranges from 26.8% to 40.8%, with a peak value of 0.85. At the optimal content of 5%, the softening coefficient reaches 0.80. The results indicate that the additive NS can markedly improve the water resistance of solidified silt. This improvement is attributed to NS, which facilitates the rapid formation of calcium sulfoaluminate hydration products. These products accelerate the hydration and hardening process of cement and enhance the solidification effect and increase the strength and water resistance of the solidified silt.

### 4.6. Freeze–Thaw Cycle

**Figure 12:**
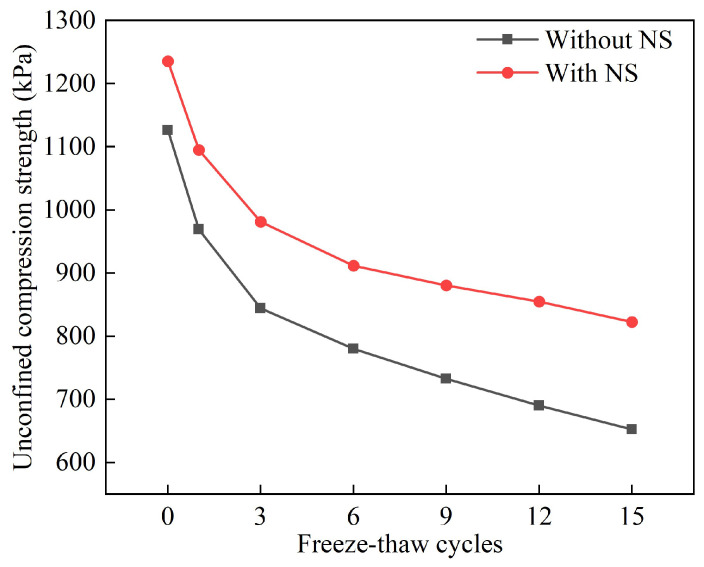
Changes in the strength of stabilized silt under 15 freeze–thaw cycles.

**Figure 13:**
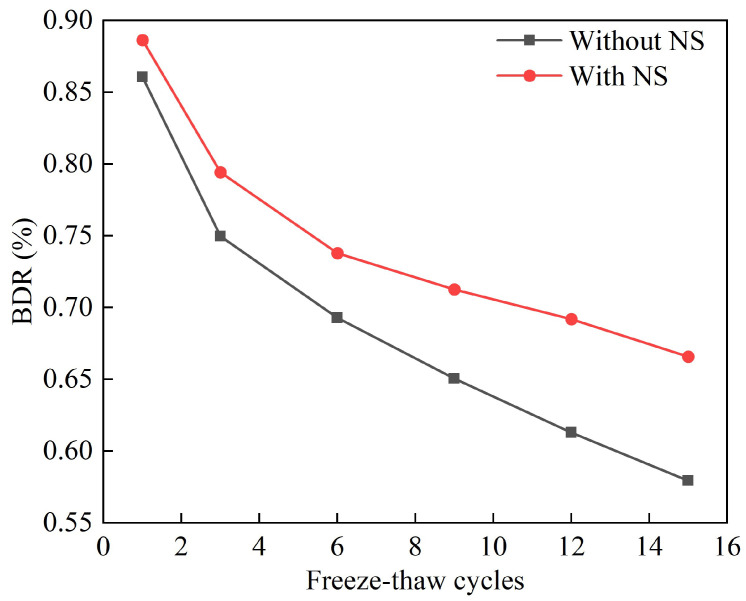
Changes in BDR of solidified silt after 15 freeze–thaw cycles.

**Figure 14:**
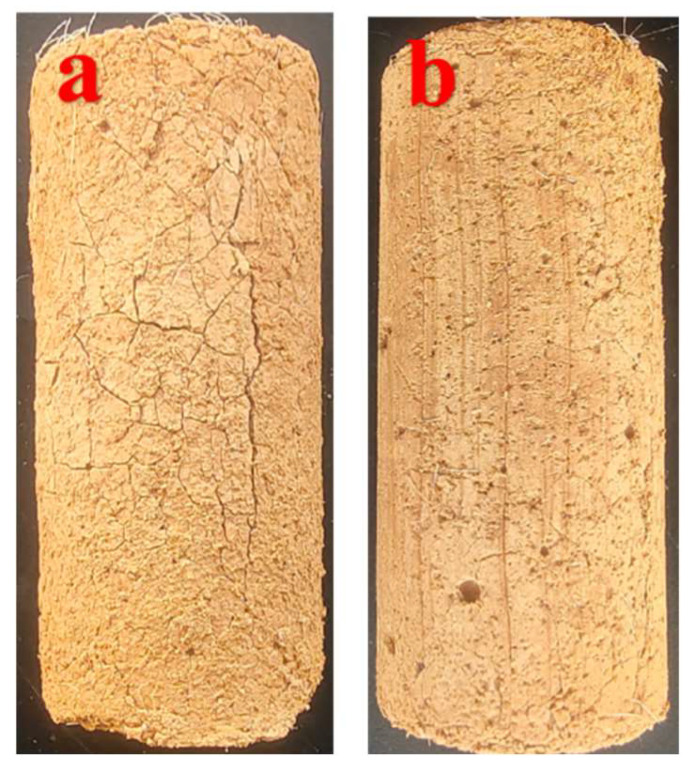
Appearance of solidified silt after 15 cycles of freeze–thaw process: (**a**) with NS; (**b**) without NS.

Figure 12 and Figure 13 show the strength and frost resistance index changes of the solidified silt after 15 freeze–thaw cycles. The analysis indicates a rapid decrease in strength and the frost resistance index in the initial phase, which was followed by a trend stabilization. Specifically, the strength of the unblended NS group dropped to 0.65 MPa after 15 freeze–thaw cycles, while the strength of the NS-blended group decreased to 0.82 MPa. Additionally, the frost resistance index for the unblended NS group fell to 57.9%, compared to a reduction of 66.6% for the NS-blended group. This clearly shows that the unblended NS group has a significantly faster rate of decline in strength and the frost resistance index than the NS-blended group. Figure 14 further reveals that after 15 freeze–thaw cycles, the samples of solidified silt from the unblended NS group exhibited noticeable cracking, as well as instances of material spalling. These results suggest that the additive NS can considerably mitigate the damage caused to the strength of solidified silt by freeze–thaw cycles.

### 4.7. Microscopic Analysis

#### 4.7.1. XRD Analysis

**Figure 15:**
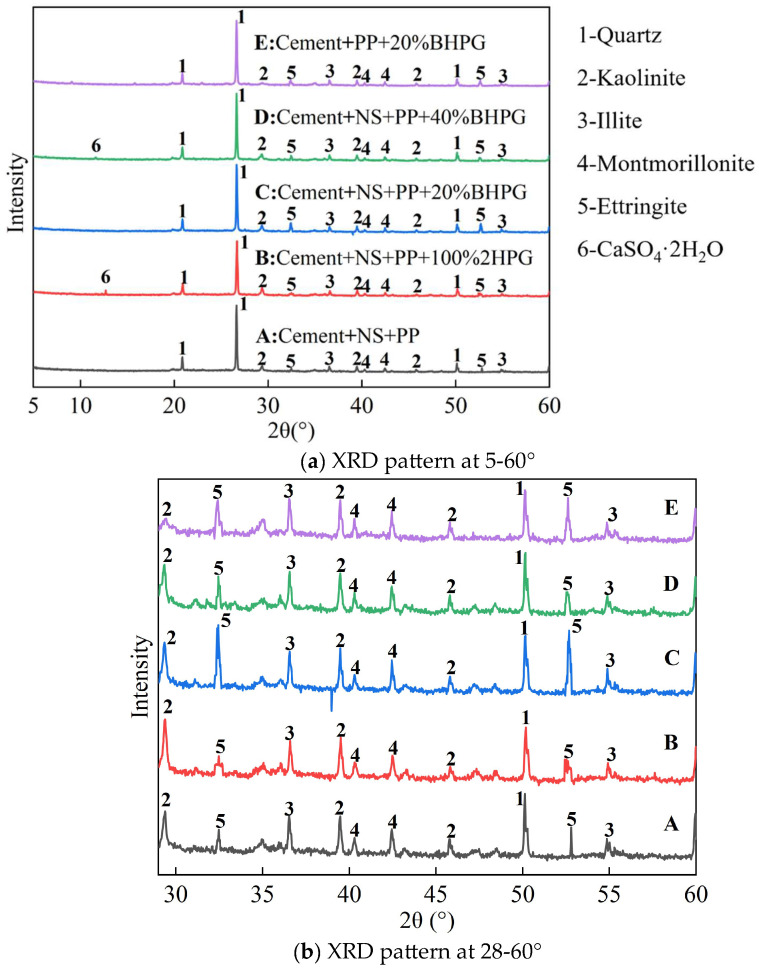
XRD pattern of solidified silt with 28-day curing period.

Figure 15 displays the XRD spectra of cured silt after 28 days. It is evident from the figure that the microscopic compositions of the samples under various conditions are largely consistent. These compositions are dominated by quartz, kaolinite, illite, montmorillonite, and ettringite (AFt). Figure 15b is a partial amplification of the XRD pattern. Compared to sample E, sample C shows a higher diffraction intensity for the AFt peak, while samples B and D exhibit peaks for gypsum dihydrate (2HPG).

The results indicate that the addition of NS significantly increases the amount of ettringite formed in the cured silt. Furthermore, the peaks of calcium sulfate dihydrate of B and D indicate that there is unreacted 2HPG in the system. This is consistent with the previous experimental findings, which demonstrate that NS promotes the formation of ettringite by supplying free sulfate ions that react with calcium ions in the cement. This reaction leads to the production of calcium sulfate, which consequently enhances ettringite formation.

#### 4.7.2. SEM Observation

**Figure 16:**
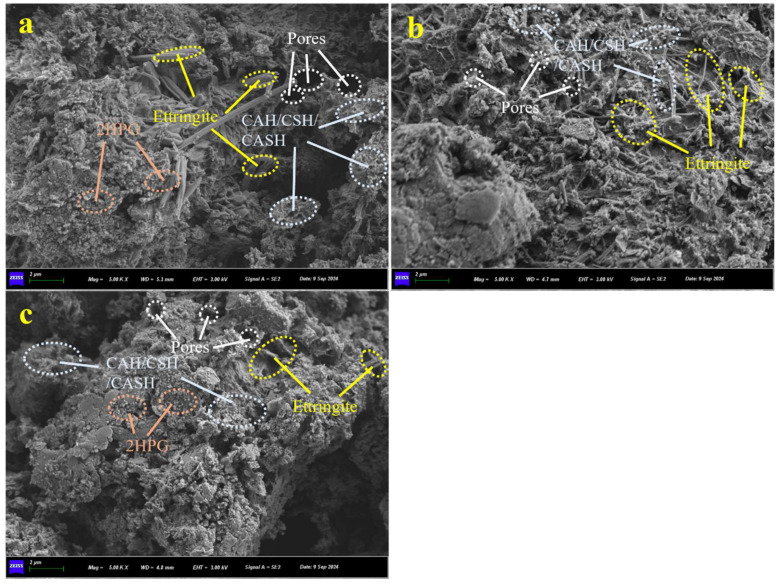
Scanning electron microscopy of solidified silt with 28-day curing period: (**a**) without NS, 20% BHPG; (**b**) with NS, 20% BHPG; (**c**) without NS, 40% BHPG.

Figure 16 shows the SEM images of cured silt after a standard curing period of 28 days. It can be observed that a substantial amount of needle-like and rod-like ettringite has formed, which fills the voids between soil particles along with hydration product (C-A-H, C-S-H, C-A-S-H) gels. Some hydration products particularly encapsulate phosphogypsum. Compared to Figure 16a, the quantity of ettringite in Figure 16b has notably increased. This increase demonstrates that NS has supplemented the sulfate ions, which in turn enhances the formation of ettringite. This is consistent with the results of the XRD pattern and further substantiates the promoting effect of NS on the stabilized silt system.

In contrast to Figure 16b,c, a higher content of BHPG is shown, with some 2HPG particles not being encapsulated by hydration products; instead, they are scattered throughout the system, negatively impacting the structure. Moreover, the number of ettringite crystals significantly decreases, and this results in an overall reduction in strength.

## 5. Conclusions

This study proposes the simultaneous treatment of muddy soil using two-phase phosphogypsum, cement, and fibers, optimizing performance by adding NS. Unconfined compressive strength tests, water resistance tests, and freeze–thaw cycle tests were conducted to investigate the influence of the content and composition proportion of two-phase phosphogypsum on the strength of the solidified silt. Additionally, XRD and SEM analyses were utilized to examine the microstructure of the solidified system. The following conclusions were drawn:

(1) Both the total content of phosphogypsum and the proportion of BHPG in two-phase phosphogypsum exhibit optimal values. Specifically, when the content of two-phase phosphogypsum is 5% and the proportion of BHPG is 20%, the unconfined compressive strength of solidified silt reaches its peak at 1.42 MPa. At the same time, the strain, stress, and deformation modulus of solidified silt are optimal. However, as the BHPG content continues to increase beyond this point, the degree of brittle damage in the solidified silt initially increases and subsequently decreases.
(2) The incorporation of NS substantially enhanced the water resistance and freeze–thaw resistance of the solidified silt. The softening coefficient improved by 26.8% to 40.8%, reaching a maximum of 0.85. The frost resistance index increased from 36.9% to 23.92%. These findings indicate that NS improves the water stability and freeze–thaw resistance of solidified silt through the supplementation of sulfate ions.
(3) When the content of BHPG in the two-phase phosphogypsum is 20%, a large number of AFt crystals are generated, significantly enhancing the strength. The addition of NS further promotes the formation of AFt, thereby improving the strength and durability of the solidified silt. However, when the contents of the two-phase phosphogypsum and BHPG are too high, the unencapsulated 2HPG particles scatter in the system. This scattering phenomenon compromises the structural integrity and ultimately reduces the strength.

This study provides innovative approaches and solutions for treating silty soil and industrial solid waste, offering significant economic and environmental benefits. In this study, the durability of long-term solidified silt needs to be tested, and field tests should be considered.

## Figures and Tables

**Table 1:** Physical properties of test soil (%).

Relative Density	Plastic Limit	Liquid Limit	Organic Matter Content
1.50	38.1	20.7	3.21

**Table 2:** Chemical composition mass fraction of phosphogypsum (%).

Composition Content	CaO	SO_3_	SiO_2_	P_2_O_5_	Al_2_O_3_	MgO	As_2_O_5_	Cr_2_O_5_	BaO	F	Crystal Water
PG	30.61	40.10	4.61	1.49	2.15	0.01	0.58	0.01	0.06	0.14	18.27

**Table 3:** Chemical composition of cement mass fraction (%).

Composition Content	CaO	SO_3_	SiO_2_	P_2_O_5_	Al_2_O_3_	MgO
OPC	62.01	4.01	21.60	0.19	5.15	1.01

**Table 4:** Physical and mechanical properties of polypropylene fiber.

Diameter/μm	Tensile Strength/MPa	Elastic Modulus/GPa	Acid–Alkali Resistance	Maximum Elongation/%	Density/(g·cm^−3^)	Melting Point /°C	Effect of Self-Dispersal
32.7	469	4.24	Extremely strong	28.4	0.91	169	Funkiness

**Table 5:** Mixture proportion of two-phase phosphogypsum solidified silt material.

Two-Phase Phosphogypsum			Silt	Admixture		
BHPG/%	2HPG/%	BHPG + 2HPG/%	Experiment Soil/%	Cement/%	PP/%	NS/%
0	100	0, 5, 10, 15, 20	100, 95, 90, 85, 80	15	0.5	7
10	90					
20	80					
30	70					
40	60					

## Data Availability

The original contributions presented in this study are included in the article. Further inquiries can be directed to the corresponding author.
